# Integration of patient-reported outcomes and myokine profiling for the detection of physical inactivity in COPD: an exploratory multicentre study

**DOI:** 10.1038/s41598-025-34436-y

**Published:** 2026-01-03

**Authors:** Tasuku Yamamoto, Tsunahiko Hirano, Keiko Doi, Ayumi Fukatsu-Chikumoto, Keiji Oishi, Koichiro Takahashi, Kazuhisa Asai, Seigo Sasaki, Masanori Nakanishi, Yoshiaki Minakata, Kazuto Matsunaga

**Affiliations:** 1 Graduate School of Medicine, Department of Respiratory Medicine and Infectious Diseases, Yamaguchi University, Ube, 755-8505 Japan; 2 Department of Respiratory Medicine, Saga University Hospital, Saga, 849-8501 Japan; 3 Graduate School of Medicine, Department of Respiratory Medicine, Osaka Metropolitan University, Osaka, 545-8585 Japan; 4 Department of Respiratory Medicine, NHO Wakayama Hospital, Mihama, 644-0044 Japan; 5 Third Department of Internal Medicine, Wakayama Medical University, Wakayama, 641- 8509 Japan

**Keywords:** Physical inactivity in COPD, Sedentary behaviour, Patient-reported outcomes, Circulating myokines, Biomarkers of physical activity, Biomarkers, Diseases, Health care, Medical research

## Abstract

Physical inactivity strongly predicts poor prognosis in chronic obstructive pulmonary disease (COPD) but is often underrecognized. We investigated whether combining patient-reported outcomes (PROs) with myokine profiling enhances detection of inactivity in COPD. In this multicentre cross-sectional study, 73 patients with stable COPD underwent PRO assessment (modified Medical Research Council dyspnea scale (mMRC), dyspnea-specific PROs (PROMs-D), COPD Assessment Test (CAT), Shortness of Breath Daily Activities Questionnaire (SOBDA-Q), and Kihon Checklist (KCL)), serum myokine measurement, and accelerometer-based physical activity evaluation, stratified into 1.0–1.5 METs (low-intensity/sedentary), ≥ 3.0 METs (moderate), total activity (METs·h), and step count. Correlation and logistic regression analyses were performed. mMRC and PROMs-D correlated negatively with moderate activity and step count. Among myokines, growth differentiation factor-15 (GDF-15), fatty acid binding protein 3 (FABP3), and brain-derived neurotrophic factor (BDNF) showed moderate associations with physical activity: GDF-15 and BDNF with low-intensity, GDF-15 with moderate, and FABP3 and BDNF with step count. Combined PRO–myokine models outperformed single markers, with areas under the curve of 0.77 for low-intensity activity, 0.82 for moderate activity, and 0.86 for step count. In conclusion, integrating PROs and myokines improves the specificity and accuracy of inactivity detection in COPD. This multidimensional strategy may facilitate early, personalized interventions.

**Supplementary Information:**

The online version contains supplementary material available at 10.1038/s41598-025-34436-y.

## Introduction

Physical inactivity is a major determinant of poor prognosis in patients with chronic obstructive pulmonary disease (COPD), increasing the risks of hospitalisation, cardiovascular events, cancer, and all-cause mortality^1–3^. Sedentary behaviour, a subset of physical inactivity, is defined as performing activities in a sitting or reclining posture with an energy expenditure of ≤ 1.5 METs^4^. Although increased sedentary behaviour does not necessarily correspond to decreased physical activity^5^, sedentary behaviour is associated with reduced physical function and decreased quality of life (QoL)^6^. In addition, previous studies have demonstrated that sedentary behaviour is an independent prognostic indicator of COPD^7^. Rehabilitation and nutritional interventions are emphasised as important strategies that can facilitate the preservation of physical activity and improvement of clinical outcomes. However, for patients with advanced COPD, who typically experience disease progression and chronic illness, complete resolution of subjective symptoms such as dyspnoea is often difficult; therefore, early and accurate identification of physical inactivity and timely initiation of interventions are essential.

Accurate and prompt detection of physical inactivity in routine clinical practice remains challenging. One major reason for this is that physical (in)activity encompasses a wide spectrum of intensity levels, from sedentary behaviour to moderate-to-vigorous activity. In addition, physical (in)activity is influenced by a complex interplay of physiological, psychological, and social factors, which makes it difficult to evaluate activity levels using a single clinical parameter or threshold.

Patient-reported outcomes (PROs) are commonly used to assess symptom burden and perceived activity limitations. Instruments designed for the assessment of PROs allow for non-invasive quantitative evaluation of subjective dimensions such as health status, symptoms, QoL, and functional capacity as experienced by patients in their daily environments. In addition, PROs are useful not only for evaluating treatment effects and adjusting therapeutic plans but also for comprehensive assessment and comparative analysis of patient conditions^8^. However, accumulating evidence suggests that PROs have limited quantitative accuracy in capturing actual physical activity. For example, in stable outpatients with COPD, nearly half of those who reported minimal dyspnoea (the modified Medical Research Council dyspnea scale (mMRC) 0–1) were found to have objectively low physical activity levels on accelerometry, indicating substantial underestimation of inactivity when relying solely on self-reported dyspnoea, likely due to consciously or unconsciously avoiding exertion and activity that would otherwise provoke symptoms^9^. Similar discrepancies have been reported in older adults, where self-reported physical activity did not consistently align with accelerometer-based measurements^10^. Furthermore, a systematic review of self-reported physical activity questionnaires demonstrated that their validity and agreement with criterion measures such as accelerometry are generally limited, partly due to recall bias, misclassification, and heterogeneity in questionnaire structure^11^. Moreover, although PROs capture behavioural or perceptual limitations, they do not reflect underlying biological changes associated with muscle disuse^12^.

Skeletal muscles and bones are not merely mechanical organs of movement; they also serve as endocrine organs. Circulating myokines, which are muscle-derived signalling molecules linked to inflammation, oxidative stress, and metabolic dysfunction, have recently emerged as promising biomarkers for objective assessment of physical inactivity. Myokines such as irisin and myostatin, which regulate skeletal muscle and bone metabolism, are dysregulated in patients with COPD and associated with reduced exercise capacity and muscle dysfunction^13–15^. Moreover, the pro-inflammatory cytokine interleukin-6 is highly expressed in patients with COPD and has been implicated in decreased muscle strength and lower physical activity levels^15,16^. Growth differentiation factor-15 (GDF-15) has been associated with reduced muscle mass and impaired exercise capacity in COPD, suggesting its potential role as an indicator of muscle dysfunction and systemic stress responses^17^. Likewise, although evidence remains limited, brain-derived neurotrophic factor (BDNF) has been reported to increase following pulmonary rehabilitation, implying a link to muscle activation and neuroplastic adaptation^18^. Fatty acid–binding protein 3 (FABP3), a marker of muscle injury and metabolic stress, has also been associated with sarcopenia and reduced physical performance in older adults^19^. Myokines alone may not fully quantify physical activity because their circulating levels are influenced by multiple extra-muscular factors. For example, GDF-15 is strongly induced by systemic inflammation^20^, cigarette smoke exposure^21,22^, comorbid cardiovascular disease^23^, malnutrition^24^, and acute physiological stress. As such, elevations in GDF-15 do not necessarily reflect muscle activity per se. These confounding influences indicate that myokines capture only part of the complex behavioural, muscular, and systemic components underlying physical inactivity.

While myokines reflect biological alterations associated with muscle disuse, PROs provide insights into behavioural avoidance, perceived symptom burden, and functional limitations experienced in daily life. Integrating these two dimensions may offer a more comprehensive and clinically meaningful evaluation of physical inactivity than either measure alone. However, the relative contribution of each myokine to different activity intensities remains unclear. In addition, whether these markers can complement PROs in identifying physically inactive individuals with COPD is not clearly understood. This study was conducted to investigate whether the combination of PROs and myokines improves the identification of reduced physical activity in patients with COPD, particularly across various objectively measured activity domains such as step count and metabolic equivalents (METs).

## Materials and methods

### Study design and participants

This was a cross-sectional study of 73 stable patients with COPD aged ≥ 40 years who were enrolled in a multicentre program conducted for the evaluation of individualised step-count goals (Effects of providing individual target step count for 6 months on the number of steps in patients with COPD, INTAR-STEP study, UMIN000046390). Participants were recruited between June 2022 and January 2023. COPD was defined as a post-bronchodilator forced expiratory volume in one second (FEV_1_) to forced vital capacity (FVC) ratio of less than 0.7. Patients with other pulmonary diseases, such as asthma or bronchiectasis, were excluded^25^. Additional exclusion criteria were as follows: (1) acute exacerbation within the past three months; (2) severely restricted physical activity due to comorbidities; (3) use of daytime oxygen therapy; and (4) deemed unsuitable for participation in the study by the principal investigator or co-investigators. As part of the baseline assessment, all participants underwent spirometry, completed multiple PRO instruments, provided serum samples, and wore a triaxial accelerometer for the assessment of physical activity.

The study protocol was approved by the Institutional Review Board of Yamaguchi University Hospital (approval number: H2022-015c). Written informed consent was obtained from all participants. All study procedures were conducted in accordance with the guidelines of the Declaration of Helsinki.

### Patient-reported outcomes

PROs were assessed using mMRC, COPD Assessment Test (CAT), Shortness of Breath Daily Activities Questionnaire (SOBDA-Q)^26^, dyspnea-specific PROs (PROMs-D)^27^, and the Kihon Checklist (KCL), a measure of a frailty screening tool developed by the Japanese Ministry of Health, Labour, and Welfare. Considering that behavioural avoidance makes it difficult to evaluate shortness of breath and dyspnoea using conventional methods, we specifically selected the SOBDA-Q and PROMs-D to enable precise assessment of subjective symptoms experienced by patients with COPD during low-intensity physical activity. The order of questionnaire administration was not predetermined or randomized.

### Measurement of myokines

Circulating myokines associated with inflammation, oxidative stress, and muscle disuse were quantified using enzyme-linked immunosorbent assays (ELISAs) and a multiplex immunoassay panel (MILLIPLEX^®^ MAP Human Myokine Magnetic Bead Panel, Millipore, USA) based on Luminex^®^ xMAP^®^ technology. GDF-15 was measured separately using an ELISA. The multiplex panel included the following 15 myokines: FABP3, osteonectin (also known as SPARC), interleukin-6 (IL-6), myostatin (growth differentiation factor-8, GDF-8), fractalkine (C-X3-C motif chemokine ligand 1, CX3CL1), BDNF, oncostatin M (OSM), erythropoietin (EPO), follistatin-like protein 1 (FSTL-1), fibroblast growth factor 21 (FGF-21), interleukin-15 (IL-15), osteocrin (musclin), irisin, apelin, and leukaemia inhibitory factor (LIF). Only myokines quantifiable in more than 50% of participants were included in the final analysis.

### Assessment of physical activity

Physical activity was objectively assessed using validated accelerometers (Active style Pro HJA-750 C^®^, Omron Healthcare Co., Ltd., Kyoto, Japan), which recorded time spent performing activities of different intensities (1.0–1.5 METs, ≥ 3.0 METs), total physical activity (PA) expressed as METs-hr, and daily step counts. Measurements and calculations were performed according to previously reported methods^28^. Participants were instructed to wear the accelerometer continuously for two weeks. The device was mounted on the waist and worn from the time participants woke up until they went to bed. Eligible days excluded those that did not match the scheduled temperature conditions, days the participants performed special activities (e.g., moving house), or days affected by COVID-19-Frelated movement restrictions. Average daily activity (activity level or activity time) was calculated as the mean value across at least three valid days.

### Statistical analysis

Spearman’s rank correlation coefficients were calculated to assess associations among PROs, circulating myokines, and physical activity indices across various intensity levels. Age- and body mass index (BMI)-adjusted correlation analyses were also performed. The participants were categorised based on the lowest quartile of each physical activity domain (bottom 25%) to identify physically inactive individuals. Regarding time spent performing 1–1.5 METs activities (sedentary time), participants in the highest quartile (top 25%) were defined as individuals with a decreased physical activity level. Regarding total physical activity, participants were divided into two groups using 1.5 METs-hr as the cutoff. Univariate logistic regression and receiver operating characteristic curve analyses were conducted to evaluate the discriminative ability of individual markers. Areas under the curve (AUC) ≥ 0.7 were considered significant. Cut-off values were determined using the Youden index. Multivariable logistic regression models were constructed using stepwise variable selection. The number of explanatory variables was determined based on the reported usefulness of models using an events-per-variable ratio of approximately 5–9^29^. To ensure model stability and validity, multicollinearity was assessed using variance inflation factors (VIF) and principal component analysis biplots, whereas model fit was evaluated using measures such as the pseudo-R-squared (U). Statistical significance was set at *p* < 0.05. All statistical analyses were performed using JMP^®^ Pro 18.1.1 (SAS Institute, Cary, USA).

## Results

### Participant characteristics and baseline measurements

Participant characteristics, physical activity levels, PROs, and myokine measurements are summarised in Table 1. The median age of the participants was 75.0 years, and 96% of them were male. The participants showed moderately impaired pulmonary function (median percent-predicted forced expiratory volume in 1 s (%FEV_1_): 71.5%). The participants also exhibited low physical activity levels, as indicated by a median daily step count of 3,777 steps and total PA of 2.84 METs-hr. PROs demonstrated substantial variability, with 44 participants scoring ≥ 10 on the CAT and 20 classified as frail according to the KCL. The myokines that were reliably quantified in more than 50% of samples are listed in Table 1, whereas those for which measurements were attempted but could not be stably obtained in majority of cases are listed in Supplementary Table S1. Of the 16 myokines tested, 8 were stably measurable and included in the final analysis.

**Table 1:** Patient characteristics, patient-reported outcomes, and physical activity levels measured using an accelerometer.

Patient characteristics	
Sample size, n (M/F)	73 (70/3)
Age (years)	75.0 (69.0–78.0)
BMI (kg/m^2^)	22.7 (20.8–24.5)
Smoking history (pack-years)	46.0 (28.9–72.0)
IC (L)	2.11 (1.85–2.44)
FEV_1_ (L)	1.82 (1.31–2.09)
%FEV_1_ (%)	71.5 (55.2–77.4)
FVC (L)	2.99 (2.61–3.55)
%FVC (%)	92.1 (81.5–103.6.5.6)
FVC/FEV_1_ (%)	57.8 (49.3–65.7)
GOLD, n (1/2/3/4)	14/45/9/5
Physical activity level	
1.0–1.5.0.5 METs (min)	393.3 (313.2–465.3.2.3)
≥ 3.0 METs (min)	48.2 (28.9–65.7)
Total-PA (METs/hr)	2.84 (1.66–3.94)
Step count (steps)	3777 (2064–5507)
PROs	
mMRC, n (grade: 0/1/2/3/4)	1 (0.0–2.0), 26/23/14/9/1
PROMs-D, n (grade: 0/1/2/3/4)	0.0 (0.0–1.0), 45/24/3/0/1
SOBDA-Q (average)	
Dietary/indoor activity/outdoor activity/ Recreation/morning/nighttime activity	6.0 (6.0–6.0)/6.0 (5.0–6.0)/6.0 (5.0–6.0)/ 5.0 (4.3–5.7)/6.0 (6.0–6.0)/6.0 (5.4–6.0.4.0)
CAT, n (total score < 10/≥10)	9.0 (4.5–12.0), 44/29
KCL, n (Robust/Prefrail/Frail)	4.0 (2.0–8.0), 30/23/20
Myokines, n (Measurable/Total)	
GDF-15 (pg/mL)	1256 (898–1742), 73/73
Irisin (pg/mL)	2.9 (2.6–3.6), 73/73
FABP3 (pg/mL)	7016(3449–9984), 59/59
Osteonectin (ng/mL)	608 (485–895), 59/59
BDNF (pg/mL)	71,504 (29002–97563), 59/59
FGF-21 (pg/mL)	161 (86–443), 54/59
Oncosttin M (pg/mL)	25.2 (11.1–59.0), 43/59
Follistatin-like Protein 1 (pg/mL)	16,260 (7836–35302), 36/59

Data are presented as number of patients or median (IQR, interquartile range) unless otherwise stated. The values for both continuous and categorical variables are presented as median (IQR) followed by the number of patients in each category. The KCL categories are defined as follows: robust, 0–3; prefrail, 4–7; frail, ≥ 8. For myokines, n indicates the number of cases with measurable values over the total number of measurements performed. Only the myokines that were stably measurable in more than half of the participants are presented.

Abbreviations: BMI, body mass index; IC, inspiratory capacity; FEV₁, forced expiratory volume in 1 s; %FEV₁, percent predicted forced expiratory volume in 1 s; FVC, forced vital capacity; %FVC, percent predicted forced vital capacity; GOLD, Global Initiative for Chronic Obstructive Lung Disease; METs, metabolic equivalents; PA, physical activity; PROs, patient-reported outcomes; mMRC, Modified Medical Research Council dyspnea scale; PROMs-D, patient-reported outcome measures for dyspnea; SOBDA-Q, Shortness of Breath Daily Activities Questionnaire; CAT, COPD Assessment Test; KCL, Kihon Checklist; GDF-15, Growth Differentiation Factor 15; FABP3, Fatty Acid Binding Protein 3; BDNF, Brain-Derived Neurotrophic Factor; FGF-21, Fibroblast Growth Factor 21.

**Fig. 1:**
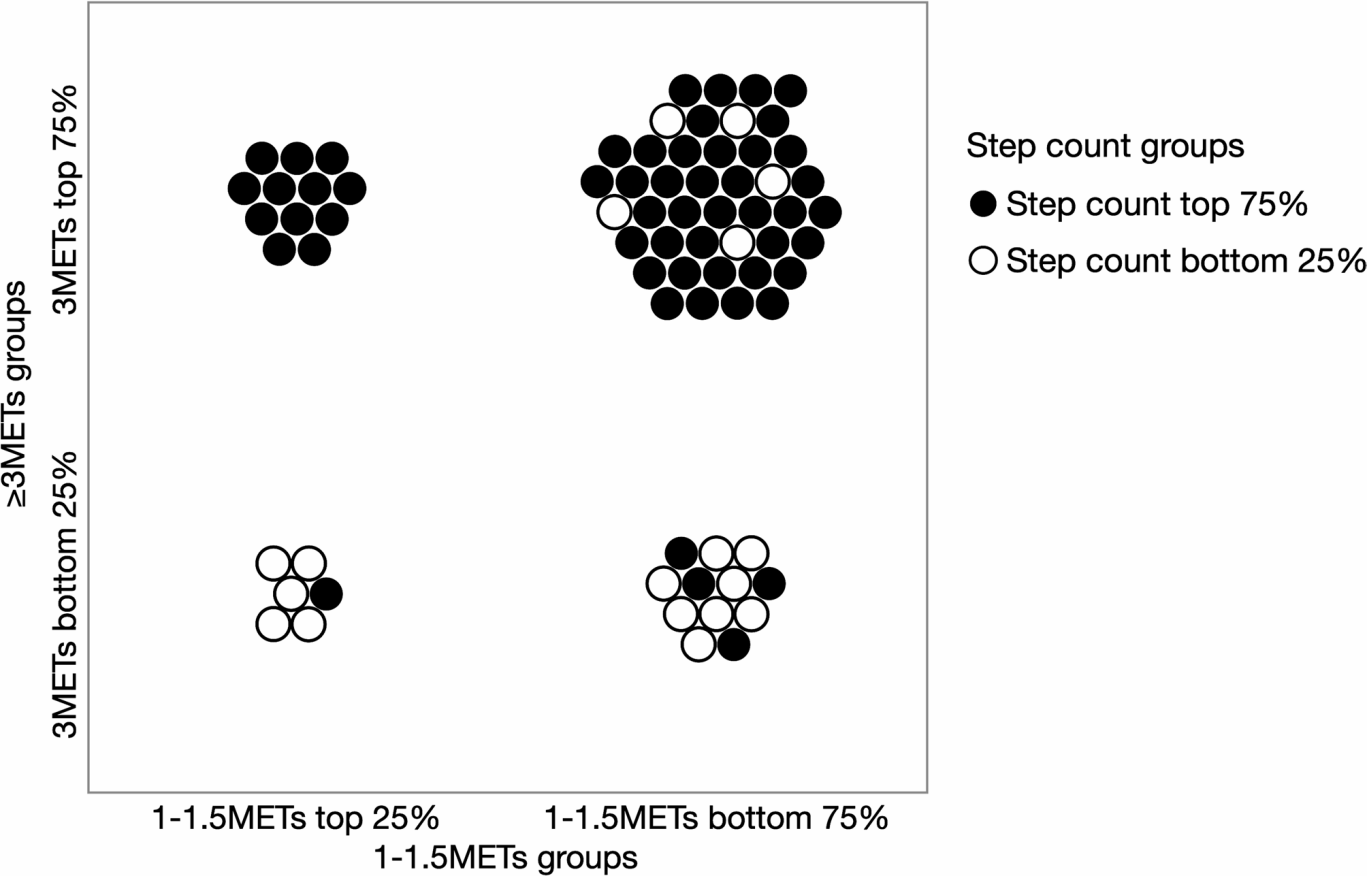
Discrepancies in the distribution of physical activity across selected measures. “1–1.5 METs top 25%” refers to the top 25% of individuals who had the longest duration of activity at this intensity, which corresponds to sedentary behaviour as defined based on accelerometer data. The sedentary population is heterogeneous and can be divided into two groups: reduced moderate-intensity activity, represented by time spent performing activities of ≥ 3 METs, and maintained moderate-intensity activity. In addition to these time-based intensity measures, step count is shown as a distinct indicator that does not directly reflect activity intensity or duration but is familiar and clinically accessible for patients. Increasing step count may help patients shift from less favourable to more favourable activity profiles. Each dot represents one patient, with filled circles indicating the top 75% in step count and open circles indicating the bottom 25%.

Figure 1 illustrates the distribution of activity time across different physical activity domains. Notably, prolonged low-intensity activity (1–1.5 METs; sedentary time) did not consistently correspond to reduced moderate-intensity activity time (≥ 3 METs), and vice versa. These findings indicate the presence of a physically “heterogeneous” group characterised by discordance across activity domains, highlighting the importance of evaluating domain-specific assessments for comprehensive characterisation of individual activity profiles.

### Associations of PROs and myokines with physical activity indices

**Fig. 2:**
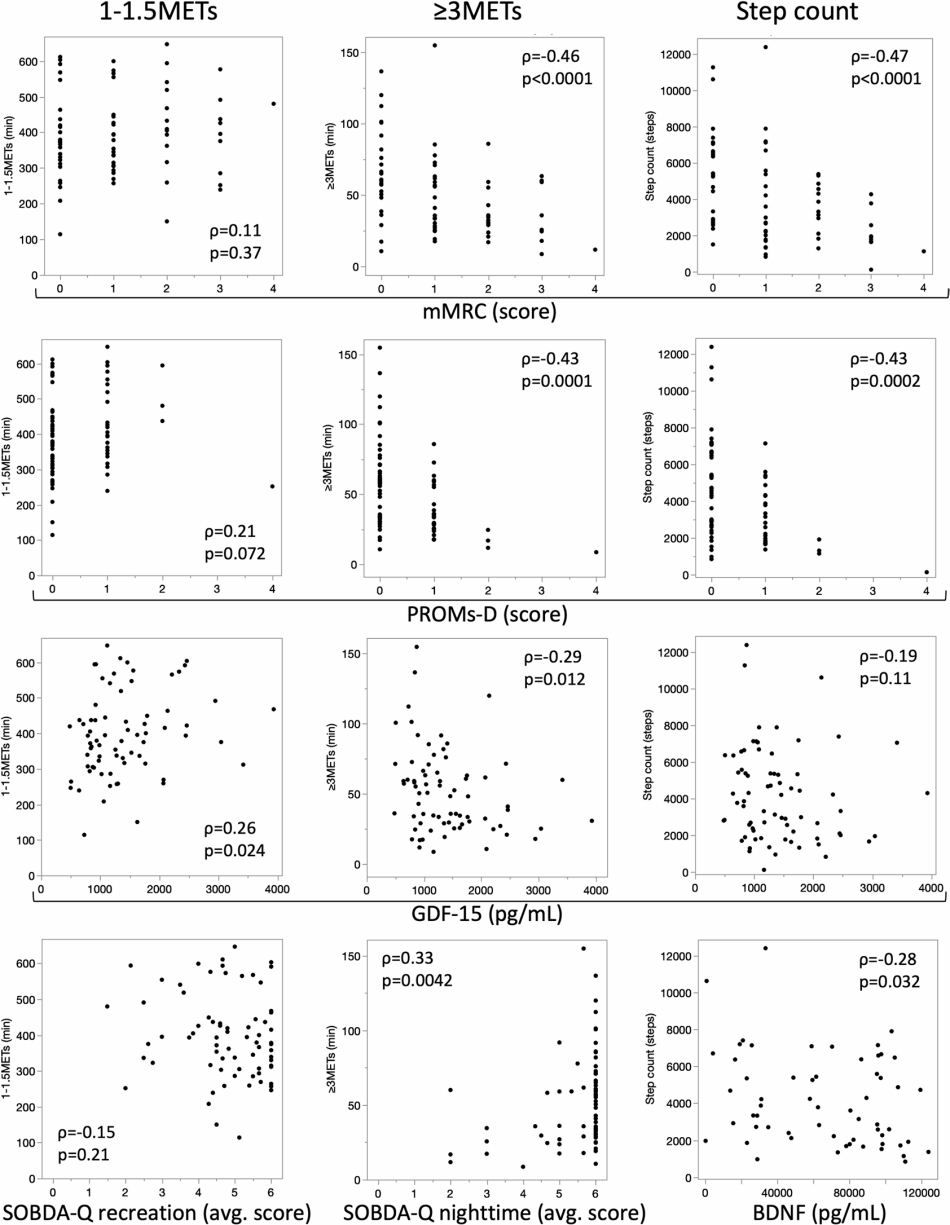
Scatterplots and Spearman correlation coefficients of the associations between biomarkers and physical activity measures. Each panel illustrates the relationship between a clinical or biological indicator and a specific physical activity measure (selected results are shown). No significant correlations were observed between sedentary-level physical activity (1.0–1.5 METs) and PROs. However, a trend was observed in the relationships between some myokines and sedentary-level activity. In contrast, physical activity at ≥ 3 METs, indicative of moderate-to-vigorous intensity, showed significant correlations with multiple PROs and myokines. In addition, step count was significantly correlated with some PROs and myokines. Correlation coefficients (ρ) represent Spearman’s rank correlation.

The correlations between PROs or myokines and physical activity indices are illustrated in Fig. 2 and Supplementary Table S2. Of the PROs, mMRC and PROMs-D showed statistically significant moderate negative correlations with moderate-intensity activity (≥ 3.0 METs; mMRC: ρ = −0.46, *p* < 0.0001; PROMs-D: ρ = −0.43, *p* = 0.0001) and step count (mMRC: ρ = −0.47, *p* < 0.0001; PROMs-D: ρ = −0.43, *p* = 0.0002). Certain SOBDA-Q domains, particularly “recreation” and “nighttime activity,” showed moderate positive correlations with moderate-intensity activity (≥ 3.0 METs; recreation: ρ = 0.43, *p* = 0.0001; nighttime: ρ = 0.33, *p* = 0.0038) and step count (recreation: ρ = 0.31, *p* = 0.0070; nighttime: ρ = 0.32, *p* = 0.0064). Total CAT and KCL scores showed a consistent negative correlation with moderate-intensity activity and step count. Sedentary-level activity showed no significant correlations with PROs; however, a trend toward a positive correlation with PROMs-D was observed (1.0–1.5 METs; ρ = 0.21, *p* = 0.072). Among the myokines, GDF-15 exhibited a distinct pattern, showing a positive correlation with sedentary activity (1.0–1.5 METs; ρ = 0.26, *p* = 0.024) and negative correlations with moderate-intensity activity (≥ 3.0 METs; ρ = −0.29, *p* = 0.012) and step count (ρ = −0.19, *p* = 0.11). BDNF and FABP3 were negatively associated with step count (BDNF: ρ = −0.28, *p* = 0.032; FABP3: ρ = −0.29, *p* = 0.024), showing patterns somewhat similar to those exhibited by GDF-15. These trends remained unchanged after adjustment for age and BMI.

### Univariate analysis of individual markers

**Fig. 3:**
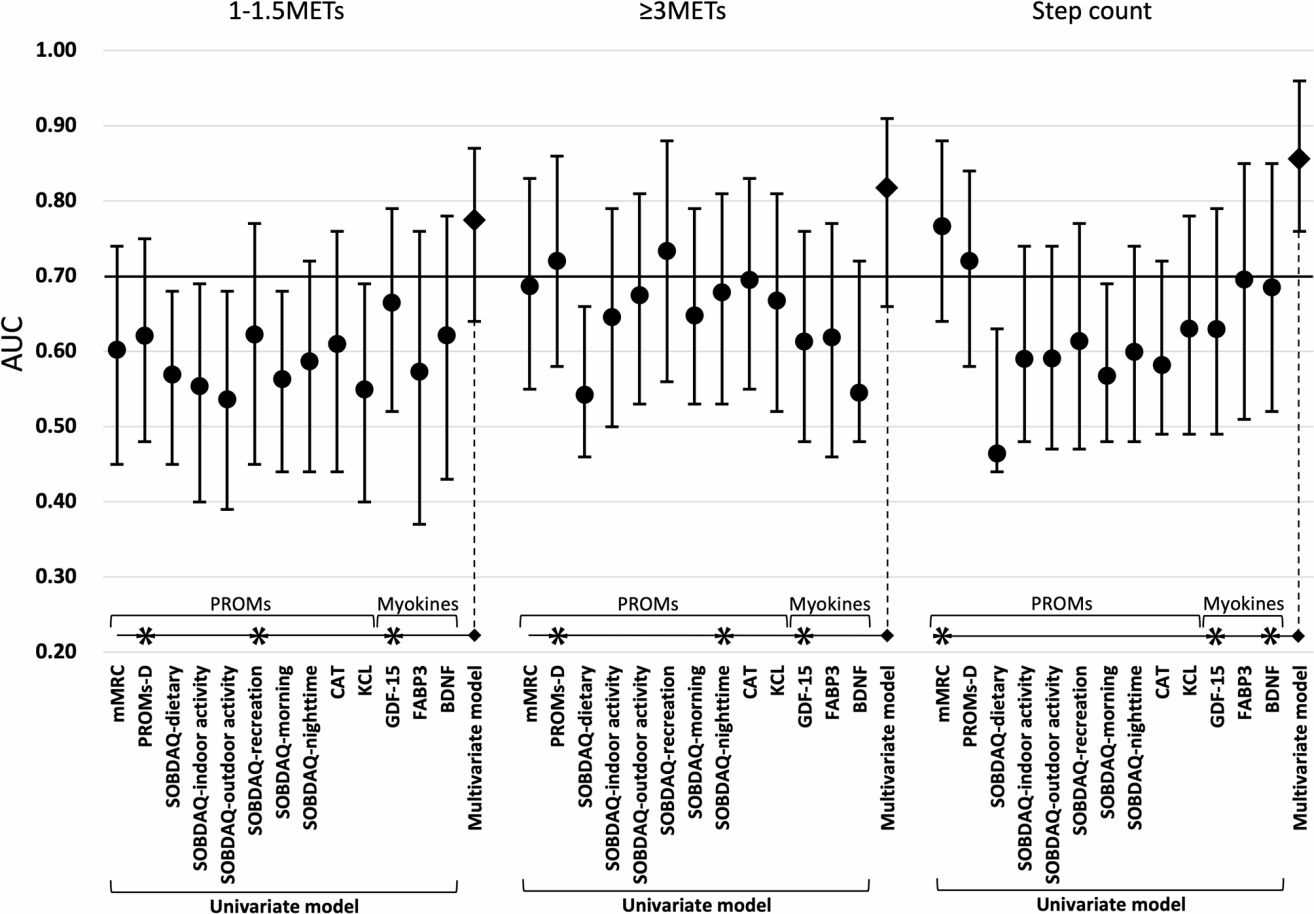
Results of univariate and multivariate logistic regression analyses. The predictive ability of each indicator for each physical activity segment was evaluated. Based on the results of the univariate analysis, selected variables were included in the multivariate models. Prolonged time spent performing sedentary-level activity (1.0–1.5 METs) was more predictive of certain myokine levels than of PROs. Conversely, reduced activity at ≥ 3 METs, representing moderate-intensity activity, was more predictive of changes in PROs than myokine levels. Combining multiple PROs and myokines improved the overall predictive performance. Asterisks indicate the variables included in the multivariate models. The 95% confidence intervals of the AUCs are shown. An AUC of 0.7 or higher was considered significant.

The discriminative performances of individual markers, assessed using univariate logistic regression, are summarised in Supplementary Table S3. A subset of these results is presented in Table 2; Fig. 3. The discriminative ability of PROs for sedentary-level activity (1.0–1.5 METs) was relatively weak; however, measures such as PROMs-D and the SOBDA-Q (recreation domain) demonstrated modest performance (AUC = 0.62; sensitivity = 0.56; specificity = 0.67; and AUC = 0.62, sensitivity = 0.44, specificity = 0.80, respectively). GDF-15 outperformed these PROs, achieving an AUC of 0.66, with a sensitivity of 1.00 and a specificity of 0.33. Regarding moderate-to-vigorous activity time, total PA, and step count, several PROs, such as the mMRC, PROMs-D, and selected SOBDA-Q domains, achieved AUC values exceeding 0.7, indicating good discriminative performance. In addition, certain myokines demonstrated moderate discriminative ability, with AUCs ranging from 0.6 to 0.7. As shown in Fig. 3, the performance of individual markers varied across activity domains.

**Table 2:** Diagnostic ability of individual PROs, myokines, and multivariable models for physical inactivity.

1–1.5.5 METs					≥ 3 METs					Step count				
Variable	AUC	Sn	Sp	cut off	Variable	AUC	Sn	Sp	Cut off	Variable	AUC	Sn	Sp	Cut off
Univariable model														
PROMs-D	0.62	0.56	0.67	1	PROMs-D	0.72	0.67	0.71	1	mMRC	0.77	0.94	0.45	1
SOBDAQ-recreation	0.62	0.44	0.80	4.3	SOBDAQ-nighttime	0.68	0.5	0.85	5	BDNF (pg/mL)	0.68	0.80	0.61	73,713
GDF-15 (pg/mL)	0.66	1.0	0.33	904.5	GDF-15 (pg/mL)	0.61	0.33	0.89	2092	GDF-15 (pg/mL)	0.63	0.89	0.33	921.2
Multivariable model	0.77	0.72	0.82	-	Multivariable model	0.82	0.72	0.84	-	Multivariable model	0.86	0.87	0.75	-

To detect reduced physical activity (bottom 25%) in each activity stratum, logistic regression analysis was performed using both univariable and multivariable models selected through stepwise selection. The results show the performance of each multivariable model and the corresponding univariable models for variables included in the multivariable models within each activity stratum. The multivariable model showed improved detection of reduced physical activity relative to the univariable model.

Abbreviations: AUC, area under the curve; Sn, sensitivity; Sp, specificity.

### Multivariable models combining PROs and myokines

**Fig. 4:**
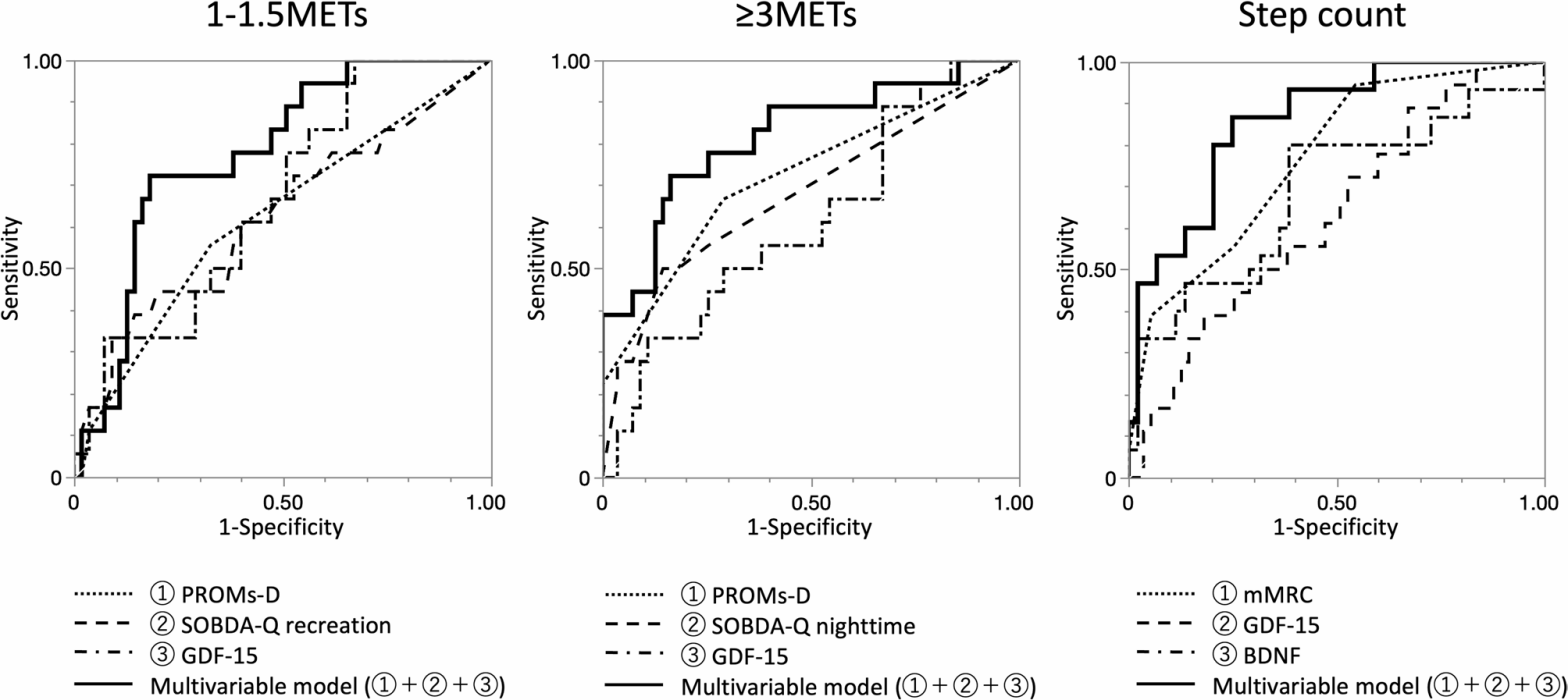
ROC curves for univariate and multivariate logistic regression models. The multivariate model, shown as a bold solid line, demonstrates improved predictive performance compared to the univariate models. For the prediction of 1.0–1.5 METs, the multivariable model demonstrated a higher AUC (0.77) than the univariate models based on PROMs-D (0.62), SOBDA-Q recreation (0.62), and GDF-15 (0.66), with corresponding differences in AUC of *p* = 0.048, 0.068, and 0.093, respectively. For ≥ 3.0 METs, the multivariable model also showed superior discriminative performance (AUC 0.82) compared with the univariate models using PROMs-D (0.72), SOBDA-Q nighttime (0.68), and GDF-15 (0.61), with AUC differences of *p* = 0.031, 0.035, and 0.028, respectively. Similarly, for step count, the multivariable model achieved the highest AUC (0.86), exceeding those of the univariate models based on mMRC (0.77), GDF-15 (0.68), and BDNF (0.63), with corresponding AUC differences of *p* = 0.10, 0.041, and 0.006, respectively.

Multivariable logistic regression models that included both PROs and myokines demonstrated superior discriminative performance compared to the univariate models. The model for step count, which included mMRC, GDF-15, and BDNF, achieved the highest AUC (0.86), with balanced sensitivity and specificity (Table 2; Fig. 4). Similar improvements were observed across other models, including those that predicted ≥ 3.0 METs and 1.0–1.5 METs activity levels. All VIF values in these models were below 5, indicating no evidence of multicollinearity. The pseudo-R-squared (U) values ranged from 0.12 to 0.30, demonstrating a certain level of explanatory power. The explanatory variables were selected through a stepwise procedure to achieve optimal model performance. Notably, GDF-15 showed consistent associations with multiple physical activity domains on its own and was included in all three prediction models.

To identify patient subgroups in whom the inclusion of GDF-15 enhances the detection of reduced physical activity, we compared the clinical characteristics of patients who were identifiable only by multivariable models incorporating GDF-15 with those who were detectable using PRO-only models. However, because of the small sample size, no statistically significant differences were observed. Nevertheless, these subgroups showed slightly more severe patient-reported symptoms as assessed by the CAT, while lung function tended to be relatively preserved (data not shown).

## Discussion

This study demonstrates that integrating PROs with circulating myokines substantially enhances the identification of physically inactive individuals with COPD. Unlike prior work that has evaluated these measures separately, the present study shows that each captures distinct and only partially overlapping dimensions of physical inactivity. As a result, their combined use provides superior discriminative performance—particularly for step count and moderate-intensity activity (≥ 3.0 METs)—beyond what either modality can achieve alone. These findings highlight a novel, biologically and clinically grounded approach to detecting low physical activity in COPD and suggest a feasible framework for improving risk stratification in settings where objective activity monitoring is impractical.

Importantly, the multivariable model for step count demonstrated the highest discriminative performance (AUC 0.86 versus 0.82 for ≥ 3.0 METs and 0.77 for 1.0–1.5 METs). Our visualisation of the combination of ≥ 3.0 METs, 1.0–1.5 METs, and step count (Fig. 1) highlighted a heterogeneous pattern of inactivity, indicating that reductions in physical activity do not occur uniformly across all domains. Although ≥ 3.0 METs and 1.0–1.5 METs reflect time-based measures categorised according to activity intensity, step count is independent of intensity or activity type and does not represent time spent performing any activity. While intensity-based metrics provide physiologically important information about energy expenditure and are linked to clinical outcomes, step count offers distinct practical advantages: it is immediately comprehensible to patients, easily self-monitored using low-cost devices, and naturally suited to goal-setting in behavioral interventions. Our study not only emphasizes the value of referring to multiple domains when assessing physical inactivity but also suggests that step-count-based screening and intervention strategies, owing to their superior predictive performance and clinical accessibility, may be particularly well suited for integration into routine COPD care.

These findings highlight the complementary roles of behavioural and biological indicators. PROs reflect patients’ perceived limitations, which may be influenced by motivation, adaptation, or psychological factors, whereas myokines may capture the physiological footprint of muscle disuse. Notably, GDF-15 and BDNF, both associated with mitochondrial dysfunction and systemic inflammation, emerged as promising biomarkers of inactivity across multiple activity domains.

Previous studies have indicated that patients with COPD show significantly reduced physical activity levels compared to healthy individuals, with a particularly pronounced decline in moderate-to-vigorous physical activity (MVPA)^30^. As a result, time spent performing moderate-intensity activity has been a primary target in the assessment of inactivity and a basis for selection of interventions. However, sedentary time has also been identified as an independent risk factor for mortality in patients with COPD, regardless of the amount of MVPA they engage in^7^, highlighting the need for multidimensional assessment of physical activity. Although clinical tools such as PRO instruments have been used in attempts to measure sedentary time, accurately capturing this aspect of inactivity has remained challenging, partly due to underestimation resulting from behavioural avoidance^31^.

Consistent with these abovementioned findings, our results demonstrated that PROs showed a particularly strong ability to detect moderate-intensity activity. In addition, the results further suggested that different PROs may have distinct strengths in identifying physical inactivity across different intensity domains. mMRC and CAT, which are widely used in clinical practice for evaluation of patients with COPD and chronic respiratory failure owing to their simplicity and feasibility, demonstrated notable usefulness in the assessment of reduced moderate-intensity activity. In addition, PROMs-D showed a significant association with moderate-intensity activity and appeared to retain some sensitivity to lower-intensity activity compared with mMRC, reflecting the primary goal of its questionnaire design. Our results also indicated that specific SOBDA-Q domains, such as the recreation and nighttime activity domains, may serve as useful items for evaluating physical activity; however, their overall sensitivity to behavioural avoidance seemed limited. KCL score showed a consistent association with moderate-intensity activity. However, as KCL encompasses not only activities of daily living but also motor function, nutritional status, cognition, and depression, interpretation of its results using total score alone is complex.

Myokines such as GDF-15 and FABP3, which are markers of oxidative stress and muscle disuse, provide physiological insight into inactivity. Although BDNF is neuroprotective, it may also reflect airway inflammation^32^. Previous studies have reported associations of various myokines including GDF-15, FABP3, and BDNF with physical activity levels or sarcopenia in patients with COPD^18,19,33^; however, attempts to use circulating myokines alone to quantify physical activity remain limited, and their utility as standalone biomarkers is not well established. These complementary features support the combined use of behavioural and biological indicators for multidimensional profiling of physical inactivity in patients with COPD. However, it should be noted that circulating levels of myokines may be influenced by confounding factors such as age, BMI, and comorbidities. For example, GDF-15 levels increase with age^33,34^, show a negative correlation with BMI (in the low BMI range)^35^, and are elevated in various age-related diseases^36–38^, including cardiovascular disease and diabetes. In the present study, adjustment for age and BMI did not substantially alter the observed associations; however, the potential impact of comorbidities could not be fully assessed. Therefore, the results should be interpreted with caution.

The consistent selection of GDF-15 across all multivariable models is an important observation. Previous studies have also suggested that GDF-15 is an important biomarker for assessing physical activity levels in patients with COPD^33,39^. Identifying patient subgroups in whom the inclusion of GDF-15 enhances the detection of reduced physical activity may have important clinical implications. However, owing to the limited sample size, the present study was unable to yield statistically significant findings in this regard. Nevertheless, these subgroups tended to report slightly more severe symptoms on the CAT, whereas lung function appeared to be relatively preserved. Further studies are warranted to address these questions.

Our findings align with the conceptual framework proposed by Wasserman et al.^40^, which emphasises integrative assessment of the cardiopulmonary and muscular systems in response to activity demand. In this context, the combined use of PROs and myokines may serve not only as a measure of physical inactivity but also as a tool for detecting imbalances or impairments across the cardiopulmonary–muscular axis. Notably, behavioural avoidance may reduce the sensitivity of PROs for physical activity, particularly at lower intensity levels, whereas biological markers such as GDF-15 and FABP3, which are linked to sarcopenia and poor prognosis in COPD^17,41^, may serve as indirect evidence of underlying physiological dysfunction.

The primary strength of this study is that the multidimensional approach adopted in our analysis may overcome the limitations of relying solely on PROs, which are susceptible to underreporting. In addition, the results indicate that the integration of biological markers could enable a more comprehensive profiling of physical inactivity, facilitating earlier identification and initiation of personalised intervention. Importantly, our findings suggest that a combination of simple PRO questionnaires and blood-based myokine measurements may function as a practical screening strategy in real-world clinical settings to identify patients at high risk for daily activity decline. Such an approach could support timely interventions—including exercise guidance or pulmonary rehabilitation—before substantial deterioration in physical activity occurs. Moreover, the minimally invasive and technically feasible nature of circulating myokine measurement supports its potential incorporation into routine clinical practice. It should be noted that the clinical tools evaluated in this study—particularly GDF-15 measurement—are not currently approved or reimbursed for routine clinical use in most healthcare systems. This limits the immediate applicability of our findings, though they provide important proof-of-concept evidence to guide future clinical translation efforts as these biomarkers become more accessible. Additionally, the PRO instruments used lack standardized cut-off values for risk stratification, requiring further validation before clinical implementation.

Despite these abovementioned strengths, this study has several limitations that should be acknowledged. First, its cross-sectional design precludes the establishment of causal inferences regarding the relationships among physical activity, PROs, and myokines. Second, the modest sample size may limit generalisability of the results, particularly to patients with milder or more severe disease phenotypes. Third, although we measured key circulating myokines, we did not assess local muscle expression or temporal dynamics over time. Additionally, while accelerometer-based activity monitoring provides objective data, it may not fully capture the qualitative aspects of physical performance or behavioural context. Furthermore, because both the PROMs-D and SOBDA-Q assess activity limitation due to dyspnoea with conceptually overlapping items, administering them within the same visit may have introduced respondent burden and potential response bias, including priming or order effects, particularly in an elderly cohort. The order of questionnaire administration was not randomised, and thus the possibility of such bias cannot be excluded. This limitation also highlights that the use of multiple overlapping PRO tools may reduce feasibility in routine clinical practice; however, this issue may be mitigated by selecting and utilising only the most informative or clinically relevant questionnaire items. Therefore, external validation of our results in longitudinal studies using larger, independent cohorts is necessary to confirm the robustness and clinical utility of our findings. In addition, further research is needed to investigate the impact of therapeutic interventions on both PROs and myokine levels and establish clinically meaningful thresholds, which will be essential for implementing this multidimensional approach in standard care.

In conclusion, this study demonstrated that assessment of the combination of PROs and selected myokines represents a feasible and informative strategy for detecting physical inactivity in patients with COPD. This integrative approach holds promise for improving risk stratification and guiding individualised care for patients with COPD.

## Supplementary Information

Below is the link to the electronic supplementary material.

Supplementary Material 1

## Data Availability

The datasets generated and/or analysed during the current study are not publicly available due to patient privacy and ethical restrictions. The Institutional Review Board of Yamaguchi University Hospital did not approve public sharing of individual patient data. However, de-identified data are available from the corresponding author upon reasonable request and with appropriate institutional approval.
