# Supplementary material for: Integration of patient-reported outcomes and myokine profiling for the detection of physical inactivity in COPD: an exploratory multicentre study
Source: Sci Rep. 2026 Jan 3;16:4403. doi: 10.1038/s41598-025-34436-y (PMC12865182; doi:10.1038/s41598-025-34436-y)
Supplement: Supplementary file 1 — Supplementary Material 1 [file 41598_2025_34436_MOESM1_ESM.docx]

**Supplementary Table S1. Summary of myokines with unreliable measurements: Number of attempts and successful detections**

| Myokines | n (Measurable/Total) |
| --- | --- |
| Epo | 15/59 |
| Osteocrin/Musclin | 13/50 |
| IL-15 | 11/50 |
| Fractalkine/CX3CL1 | 9/63 |
| Apelin | 7/50 |
| IL-6 | 5/50 |
| Myostatin/GDF-8 | 5/50 |
| LIF | 4/50 |

Epo, erythropoietin; IL-15, interleukin-15; CX3CL1, chemokine (C-X3-C motif) ligand 1; IL-6, interleukin-6; GDF-8, growth differentiation factor 8; LIF, leukemia inhibitory factor

**Supplementary Table S2. Associations between physical activity strata and PROs/myokines**

|  | 1-1.5 METs | | ≥3 METs | | Total-PA | | Step count | |
| --- | --- | --- | --- | --- | --- | --- | --- | --- |
|  | ρ | p value | ρ | p value | ρ | p value | ρ | p value |
| PROs |  |  |  |  |  |  |  |  |
| mMRC | 0.11 | 0.37 | -0.46 | <0.0001 | -0.46 | <0.0001 | -0.47 | <0.0001 |
| PROMs-D | 0.21 | 0.072 | -0.43 | 0.0001 | -0.43 | 0.0002 | -0.43 | 0.0002 |
| SOBDA-Q |  |  |  |  |  |  |  |  |
| Dietary | -0.076 | 0.52 | 0.17 | 0.14 | 0.18 | 0.13 | 0.077 | 0.52 |
| Indoor activity | -0.018 | 0.88 | 0.30 | 0.014 | 0.30 | 0.015 | 0.27 | 0.031 |
| Outdoor activity | -0.026 | 0.83 | 0.31 | 0.0082 | 0.30 | 0.011 | 0.24 | 0.041 |
| Recreation | -0.15 | 0.21 | 0.43 | 0.0001 | 0.43 | 0.0001 | 0.31 | 0.0070 |
| Morning | 0.027 | 0.82 | 0.30 | 0.011 | 0.30 | 0.010 | 0.23 | 0.049 |
| Nighttime | -0.15 | 0.20 | 0.33 | 0.0042 | 0.33 | 0.0038 | 0.32 | 0.0064 |
| CAT | 0.17 | 0.14 | -0.29 | 0.013 | -0.28 | 0.015 | -0.27 | 0.022 |
| KCL | 0.091 | 0.44 | -0.46 | <0.0001 | -0.48 | <0.0001 | -0.34 | 0.0034 |
| Myokines |  |  |  |  |  |  |  |  |
| GDF-15 | 0.26 | 0.024 | -0.29 | 0.012 | -0.30 | 0.010 | -0.19 | 0.11 |
| FABP3 | -0.19 | 0.16 | -0.21 | 0.11 | -0.23 | 0.078 | -0.29 | 0.024 |
| BDNF | -0.26 | 0.048 | -0.15 | 0.26 | -0.15 | 0.26 | -0.28 | 0.032 |

Univariate analysis was conducted using Spearman’s rank correlation coefficients. Only variables that remained significantly correlated after adjustment for age and BMI (data not shown) are presented. The symbol ρ denotes Spearman’s rank correlation coefficients.

**Supplementary Table S3. Results of logistic regression analysis of the ability of univariable and multivariable models to detect reduced physical activity (bottom 25%) in each stratum (across all data)**

|  | 1-1.5 METs | | | | ≥3 METs | | | | Total-PA | | | | Step count | | | |
| --- | --- | --- | --- | --- | --- | --- | --- | --- | --- | --- | --- | --- | --- | --- | --- | --- |
|  | AUC | Sn | Sp | cut off | AUC | Sn | Sp | cut off | AUC | Sn | Sp | cut off | AUC | Sn | Sp | cut off |
| Univariable model |  |  |  |  |  |  |  |  |  |  |  |  |  |  |  |  |
| PROs |  |  |  |  |  |  |  |  |  |  |  |  |  |  |  |  |
| mMRC | 0.60 | 0.50 | 0.73 | 2 | 0.69 | 0.33 | 0.93 | 3 | 0.74 | 0.6 | 0.74 | 2 | 0.77 | 0.94 | 0.45 | 1 |
| PROMs-D | 0.62 | 0.56 | 0.67 | 1 | 0.72 | 0.67 | 0.71 | 1 | 0.76 | 0.73 | 0.71 | 1 | 0.72 | 0.67 | 0.71 | 1 |
| SOBDA-Q |  |  |  |  |  |  |  |  |  |  |  |  |  |  |  |  |
| Dietary | 0.57 |  |  |  | 0.54 |  |  |  | 0.53 |  |  |  | 0.46 |  |  |  |
| Indoor activity | 0.55 |  |  |  | 0.65 | 0.53 | 0.75 | 5 | 0.68 | 0.57 | 0.75 | 5 | 0.59 |  |  |  |
| Outdoor activity | 0.54 |  |  |  | 0.68 | 0.61 | 0.68 | 5.5 | 0.66 | 0.40 | 0.88 | 4.67 | 0.59 |  |  |  |
| Recreation | 0.62 | 0.44 | 0.80 | 4.3 | 0.73 | 0.50 | 0.95 | 3.75 | 0.74 | 0.53 | 0.93 | 3.75 | 0.61 | 0.35 | 0.95 | 3 |
| Morning | 0.56 |  |  |  | 0.65 | 0.44 | 0.83 | 5.67 | 0.66 | 0.47 | 0.83 | 5.67 | 0.57 |  |  |  |
| Nighttime | 0.59 |  |  |  | 0.68 | 0.5 | 0.85 | 5 | 0.71 | 0.53 | 0.84 | 5 | 0.60 | 0.39 | 0.82 | 5 |
| CAT | 0.61 | 0.33 | 0.89 | 16 | 0.69 | 0.83 | 0.51 | 15 | 0.67 | 0.47 | 0.86 | 15 | 0.58 |  |  |  |
| KCL | 0.55 |  |  |  | 0.67 | 0.72 | 0.65 | 5 | 0.68 | 0.73 | 0.64 | 5 | 0.63 | 0.61 | 0.62 | 5 |
| Myokines |  |  |  |  |  |  |  |  |  |  |  |  |  |  |  |  |
| GDF-15 | 0.66 | 1.0 | 0.33 | 904.5 | 0.61 | 0.33 | 0.89 | 2092 | 0.59 |  |  |  | 0.63 | 0.89 | 0.33 | 921.2 |
| FABP3 | 0.57 |  |  |  | 0.62 | 0.75 | 0.49 | 6168.3 | 0.59 |  |  |  | 0.70 | 0.87 | 0.52 | 6168.3 |
| BDNF | 0.62 | 0.53 | 0.80 | 31228 | 0.55 |  |  |  | 0.58 |  |  |  | 0.68 | 0.80 | 0.61 | 73713 |
|  |  |  |  |  |  |  |  |  |  |  |  |  |  |  |  |  |
| Multivariable model | 0.77 | 0.72 | 0.82 | - | 0.82 | 0.72 | 0.84 | - | 0.78 | 0.80 | 0.74 | - | 0.86 | 0.87 | 0.75 | - |

AUC, area under the curve; Sn, sensitivity; Sp, specificity

Sensitivity and specificity are reported for variables with an AUC of 0.6 or higher, considered to indicate significance. The multivariable model included the following variables: for 1–1.5 METs, PROMs-D, SOBDA-Q (indoor), and GDF-15; for ≥3 METs, PROMs-D, SOBDA-Q (nighttime), and GDF-15; for Total-PA, PROMs-D, SOBDA-Q (recreation, nighttime); for Step count, mMRC, GDF-15, and BDNF.
